# Risk of second primary lung cancer among cancer survivors stratified by the site of first primary cancer and the lung cancer screening eligibility status

**DOI:** 10.1002/ijc.35452

**Published:** 2025-04-18

**Authors:** Sara Nofal, Edwin J Ostrin, Jianjun Zhang, Jia Wu, Paul Scheet, Mara B. Antonoff, John V Heymach, Iakovos Toumazis

**Affiliations:** ^1^ Department of Health Services Research The University of Texas MD Anderson Cancer Center Houston Texas USA; ^2^ Department of General Internal Medicine The University of Texas MD Anderson Cancer Center Houston Texas USA; ^3^ Department of Thoracic/Head & Neck Medical Oncology The University of Texas MD Anderson Cancer Center Houston Texas USA; ^4^ Department of Imaging Physics The University of Texas MD Anderson Cancer Center Houston Texas USA; ^5^ Department of Epidemiology The University of Texas MD Anderson Cancer Center Houston Texas USA; ^6^ Department of Thoracic and Cardiovascular Surgery The University of Texas MD Anderson Cancer Center Houston Texas USA

**Keywords:** cancer survivors, lung cancer, lung cancer screening guidelines, personal history of cancer, risk factors

## Abstract

Personal history of cancer is an independent risk factor for developing lung cancer. However, it is not considered in the current US lung cancer screening (LCS) guidelines. In this study, we assessed the risk of developing lung cancer among cancer survivors across 24 different sites of first primary cancer stratified by their LCS eligibility status. Using data from the Patient History Database at the University of Texas MD Anderson Cancer Center, we calculated and compared the cumulative incidence of second primary lung cancer, the overall and the LCS eligibility status‐specific, stratified by the site of first primary cancer among cancer survivors. We found that among lung, head and neck (H&N), bladder, cervical, breast, and prostate cancer survivors, the risks of second primary lung cancer were statistically significantly higher compared to the overall risk among all cancer survivors (i.e., all cancer sites combined). Risk ratios (RR) ranged between 1.14 (95%CI:1.00–1.28, *p* = 0.0431) among prostate cancer survivors to 2.9 (95%CI:2.58–3.26, *p* < 0.001) among H&N cancer survivors. Other than first primary lung cancer (RR: 1.33; 95%CI:1.14–1.57; *p* < 0.001), H&N (RR: 1.73; 95%CI:1.45–2.05; *p* < 0.001) and bladder (RR: 1.32; 95%CI:1–1.74; *p* = 0.0483) cancer survivors, who were non‐eligible for LCS, had significantly higher lung cancer risk than all cancer survivors. In conclusion, H&N, bladder, cervical, breast, and prostate cancer survivors have a high risk of developing second primary lung cancer. Specifically, personal history of H&N and bladder cancer, even among non‐eligible for LCS individuals, remain at a sufficiently high risk, which warrants further consideration as an independent eligibility factor for LCS guidelines.

## Graphical abstract

What's new?

Personal history of cancer is an independent risk factor of developing lung cancer but is omitted from screening guidelines. In this study, we assessed lung cancer risk among cancer survivors across different sites of first primary cancer stratified by individuals' lung cancer screening eligibility status. We found that H&N and bladder cancer survivors non‐eligible for lung cancer screening are at high risk of developing second primary lung cancer and could benefit from lung cancer screening.

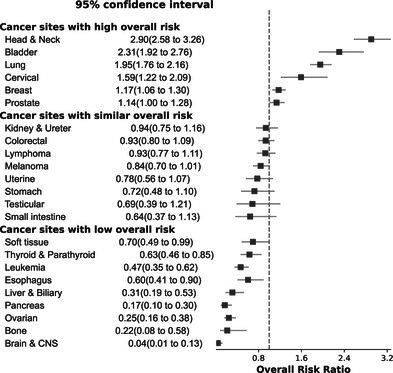

**Abbreviations**
CIs: confidence intervals
CNS: central nervous system
H&N: head & neck
LCS: Lung cancer screening
LDCT: Low dose computed tomography
MDA: MD Anderson Cancer Center
PHDB: Patient History Database
PLCO: Prostate, Lung, Colorectal, and Ovarian Screening Trial
RR: Risk ratio
USPSTF: US Preventive Services Task Force

## 1 INTRODUCTION

Lung cancer is the leading cause of cancer‐related mortality in the US and worldwide, and one of the most common second malignancies diagnosed among cancer survivors.^1^ Low dose computed tomography (LDCT) has been an effective screening tool for lung cancer, resulting in significant reduction of its mortality rate.^2^, ^3^ The 2021 US Preventive Services Task Force (USPSTF) updates for lung cancer screening recommend annual screening for individuals aged 50–80 years who have at least 20 pack‐years of smoking history, and currently smoke or have quit smoking within 15 years.^4^ The existing guidelines rely on age and smoking history without considering other important risk factors, such as personal history of cancer.^5^

The overall cancer survival rate has drastically increased from 49% in the mid‐1970s to 69% during 2013–2019 due to earlier detection and advances in treatment.^6^ As of January 1, 2022, there were over 18 million US cancer survivors. This estimate is projected to increase to 22 million by 2030.^7^ Personal history of cancer has been identified as an important independent risk factor for lung cancer.^5^, ^8^, ^9^, ^10^, ^11^ Participants of the Prostate, Lung, Colorectal, and Ovarian (PLCO) Screening Trial control arm with personal history of cancer had significantly higher odds of developing second primary lung cancer within 6 years compared to those without personal history of cancer.^9^ Patients with head and neck (H&N),^12^, ^13^ breast cancer,^14^ and Hodgkin's lymphoma^15^ were found to have increased risk of second primary lung cancer. Nevertheless, prior studies did not examine the risk of second primary lung cancer based on the individual's lung cancer screening eligibility. Therefore, it is unknown whether the current screening guidelines capture those cancer survivors with increased risk of second primary lung cancer based on the established eligibility criteria.

In our previous study, we examined the risk of developing a second primary lung cancer among H&N cancer survivors stratified by the lung cancer screening eligibility status.^13^ H&N cancer survivors who did not meet the 2021 USPSTF eligibility criteria had an increased risk of developing second primary lung cancer. Moreover, using data from the SEER cancer registry, we showed that individuals with a personal history of bladder and esophageal cancers, among others, had an elevated risk of developing lung cancer.^13^ However, we were not able to stratify individuals with a personal history of cancer by their lung cancer screening eligibility status; thus, the utility of lung cancer screening among cancer survivors by site of first primary cancer is not known.

This study examines the risk of developing second primary lung cancer among cancer survivors who received care at the University of Texas MD Anderson Cancer Center (MDA), stratified by 24 different first primary cancer sites and discusses the utility and potential implications on the 2021 USPSTF lung cancer screening guidelines.

## 2 METHODS

### 2.1 Study design and data sources

This is a retrospective secondary data analysis of the MDA Patient History Database (PHDB).

### 2.2 Study population and data collection

The PHDB is an institutional database at MDA that was launched by the Department of Epidemiology in 1999 and serves as a core institutional resource to collect standardized epidemiological information for all new patients at MDA. Data includes demographics, exposures to potential carcinogens, tobacco and alcohol use history, family cancer history, past medical and cancer history, comorbidities, and quality of life indices. Quality control checks are conducted regularly to maintain the integrity of the data. We analyzed the MDA PHDB population of cancer survivors with primary cancer diagnoses of H&N, thyroid and parathyroid, bladder, cervical, uterine, lymphoma, leukemia, melanoma, ovary, pancreas, liver and biliary, uterine, prostate, testicular, kidney and ureter, breast, colorectal, esophageal, stomach, small intestine, bone, soft tissue, brain and central nervous system (CNS), or lung, from 1999 to 2014. We included all patients with histopathologically confirmed malignant tumors of these types.

**FIGURE 1:**
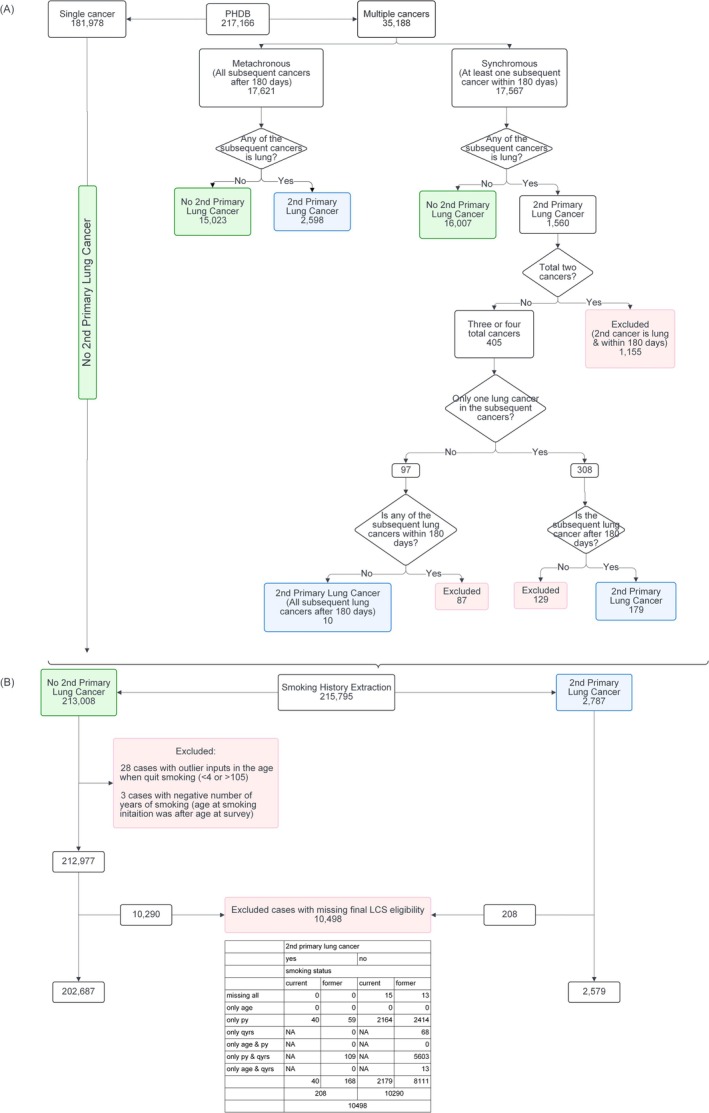
CONSORT flowchart of study sample. (A) Ascertainment of the sample stratification based on the development of second primary lung cancer. (B) Ascertainment of lung cancer screening eligibility status.

We ascertained our study population based on the total number of primary cancers and the development of second primary lung cancer by asking a series of stepwise questions (Figure 1A,B). We divided our study population into patients diagnosed with a single cancer in their lifetime and patients with multiple cancer diagnoses. Among patients with multiple cancers, we then divided the subset based on the presence of synchronous or metachronous cancers to minimize potential misclassification between metastatic and second primary lung cancer.^16^ We excluded patients with second lung cancer diagnosed within 180 days of the first primary cancer, as these were deemed to represent synchronous lung cancers (*n* = 1371). Among patients with no second primary lung cancers, we excluded 31 patients whose charts contained unreliable inputs for smoking history. For example, we excluded patients with an input age at smoking cessation younger than 4 years or older than 105 years. We also excluded 10,498 patients with insufficient age and/or smoking history to ascertain their lung cancer screening eligibility.

### 2.3 Study variables

We defined second primary lung cancers as metachronous lung cancers diagnosed at least 180 days after the diagnosis of the first primary cancer, following the criteria of Warren and Gates.^16^, ^17^ We determined the eligibility for lung cancer screening among MDA cancer survivors based on the 2021 USPSTF recommendations as follows: (1) aged 50–80 years old, (2) smoked at least 20 pack‐years, and (3) if a former smoker, quit smoking within 15 years. The individual must have met all three criteria to be eligible for lung cancer screening. We assessed the individual's screening eligibility for the period between the diagnosis of the first primary cancer through the last date of contact or the diagnosis of the second primary lung cancer, whichever occurred first. In addition to age at the diagnosis of the first primary cancer and the diagnosis of the second primary lung cancer, we reported sex and race and ethnicity of the patients.

Pack‐years variable was defined as the number of years of smoking multiplied by the number of cigarette packs smoked on average each day during the time they smoked. For people who quit smoking, we calculated the years of smoking based on the duration between smoking initiation and cessation. For people who were actively smoking, the calculation was based on the duration between smoking initiation and the last date of contact or the date of second lung cancer diagnosis. Years since quitting smoking was defined as the interval between the date of smoking cessation till the last date of contact or the date of second lung cancer diagnosis.

### 2.4 Statistical analysis

We calculated the cumulative incidence of second primary lung cancer for each first primary cancer site. We reported risk ratios (RRs) and 95% confidence intervals (CIs) to compare the cumulative incidence of second primary lung cancer among MDA cancer survivors for every primary cancer site (the overall and the stratified risk based on lung cancer screening eligibility) against the overall risk of second primary lung cancer among all MDA cancer survivors (that is, all cancer sites combined). In addition, we calculated RRs and 95% CIs to compare the cumulative incidence of developing second primary lung cancer among MDA cancer survivors who are not eligible for lung cancer screening against MDA cancer survivors who are eligible for lung cancer screening, within each primary cancer site. We also compared the cumulative incidence of second primary lung cancer stratified by its histology among MDA cancer survivors non‐eligible for lung cancer screening. Finally, we reported the proportions of MDA cancer survivors who developed second primary lung cancer within and after 5 years from their first primary cancer diagnosis, which is the commonly used surveillance period,^18^ stratified by their eligibility status for lung cancer screening. Two‐tailed Chi‐square tests were conducted with a *p*‐value of 0.05 or lower used to determine the statistical significance of the reported risk ratios. All statistical analyses were performed in Python 3.0.

## 3 RESULTS

### 3.1 Characteristics of the MDA study population

A total of 205,266 cancer survivors met the inclusion criteria (Table 1). There were 24 different sites of the primary cancer. The most frequent cancer sites were breast (15%), followed by prostate (11%), lung (9%), and colon and rectum (7%, Figure S1). About 15% of MDA cancer survivors were eligible for lung cancer screening at least for a period of time between their first primary cancer diagnosis and last date of contact. The majority were non‐Hispanic White (79%), followed by Hispanic (10%) and non‐Hispanic Black (7%) (Table 1). About 1.3% of MDA cancer survivors developed second primary lung cancer. While the mean age at the diagnosis of the first primary cancer was 56 ± 14 years, the mean age at the diagnosis of second primary lung cancer was 67 ± 10. It is important to note that among individuals who developed second primary lung cancer, about 51.4% (1325/2579) were not eligible for lung cancer screening. Of those individuals, about 77% were non‐eligible for lung cancer screening due to their smoking history not meeting the 2021 USPSTF criteria and the remaining 23% were diagnosed with second primary lung cancer at an age either <50 years or more than 80 years (Table 1).

**TABLE 1:** Rate of developing second primary lung cancer among MD Anderson cancer survivors.

	Screening Non‐eligible				Screening Eligible					
	No second lung cancer		Second lung cancer		No second lung cancer		Second lung cancer		Total	
Variables	*N*	%	*N*	%	*N*	%	*N*	%	*N*	%
All subject	173,991	99.0	1325	1.0	28,696	95.8	1254	4.2	205,266	100
Sex										
Female	89,937	51.7	659	49.7	10,694	37.3	536	43	101,826	50
Male	84,054	48.3	666	50.3	18,002	62.7	718	57	103,440	50
Race/Ethnicity										
NH White	134,833	77.5	1136	85.7	24,802	86.4	1110	89	161,881	78.9
NH Black	13,005	7.5	71	5.4	1898	6.6	84	6.7	15,058	7.3
Hispanic	19,169	11.0	74	5.6	1570	5.5	47	4	20,860	10.2
Other/Unknown	6984	4.0	44	3.3	426	1.5	13	1.0	7467	3.6
Age at 1st cancer diagnosis										
Mean (SD)	55 (15)		61 (13)		61 (8)		61 (10)		56 (14)	
Median	56		62		61		62		57	
0–39 years	24,448	14.1	85	6.4	128	0.4	31	2.5	24,692	12.0
40–49 years	32,761	18.8	144	10.9	566	2.0	96	7.7	33,567	16.4
50–59 years	41,953	24.1	264	19.9	10,229	35.6	336	26.8	52,782	25.7
60–69 years	41,726	24.0	432	32.6	11,970	41.7	509	40.6	54,637	26.6
70–79 years	24,590	14.1	323	24.4	5435	18.9	259	20.7	30,607	14.9
80+ years	8513	4.9	77	5.8	368	1.3	23	1.8	8981	4.4
Age at 2nd primary lung cancer diagnosis										
Mean (SD)			67 (11)				67 (8)		67 (10)	
Median			69				67		68	
0–39 years			16	1.2			0	0.0	16	0.6
40–49 years			81	6.1			0	0.0	81	3.1
50–59 years			171	12.9			189	15.1	360	14.0
60–69 years			376	28.4			503	40.1	879	34.1
70–79 years			471	35.5			473	37.7	944	36.6
80+ years			210	15.8			89	7.1	299	11.6

### 3.2 Overall risk of second primary lung cancer stratified by cancer sites

**FIGURE 2:**
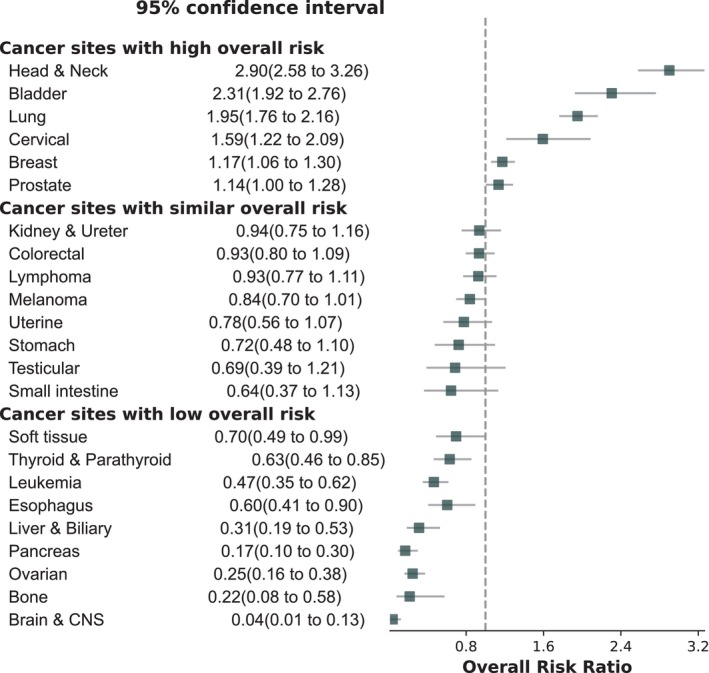
Risk of second lung cancer diagnosis by first primary cancer site relative to the overall risk (i.e., all cancers combined).

Among lung, H&N, bladder, cervical, breast, and prostate cancer survivors, the risks of second primary lung cancer were statistically significantly higher compared to the overall risk among all cancer survivors (i.e, all cancer sites combined, Figure 2). Risk ratios (RR) ranged between 1.14 (95%CI:1.00–1.28, *p* = 0.0431) among prostate cancer survivors to 2.9 (95%CI:2.58–3.26, *p* < 0.001) among H&N cancer survivors. On the other hand, among soft tissue, thyroid & parathyroid, leukemia, esophagus, liver & biliary, pancreas, ovarian, bone, and brain & CNS cancer survivors, the risks of second primary lung cancer were significantly lower compared to the overall risk among all cancer survivors. Risk ratios ranged between 0.04 (95%CI:0.01–0.13, *p* < 0.001) among brain & CNS cancer survivors to 0.7 (95%CI:0.49–0.99, *p* < 0.001) among soft tissue cancer survivors. Lastly, among kidney & ureter, colorectal, lymphoma, melanoma, uterine, stomach, testicular, and small intestine cancer survivors, the risks of second primary lung cancer were slightly lower but not significantly different from the overall risk among all cancer survivors.

### 3.3 Risk of second primary lung cancer based on lung cancer screening eligibility within each site of first primary cancer

The overall risk of developing second primary lung cancer among all cancer survivors (all cancer sites combined) who were eligible for lung cancer screening was statistically significantly higher than those cancer survivors who were non‐eligible for lung cancer screening (RR: 5.54, 95%CI:5.13–5.98, *p* < 0.001). When each cancer site was examined separately, similar results were found (Table S1). The smallest risk ratios between lung cancer screening eligible and non‐eligible groups were among lung (RR:2.00, 95%CI:1.64–2.42, *p* < 0.001), H&N (RR:3.60, 95%CI:2.88–4.49, *p* < 0.001), and bladder (RR:3.83, 95%CI:2.69–5.47, *p* < 0.001) cancer survivors. The highest risk ratios between lung cancer screening eligible and non‐eligible groups were observed among individuals with testicular cancer (RR:29.96, 95%CI:10.02–90), followed by lymphoma (RR:12.67, 95%CI:8.84–18.15), leukemia (RR:11.6, 95%CI:6.68–20.14), breast (RR:8.78, 95%CI:7.23–10.66), and stomach cancer (RR:8.42, 95CI%:3.67–19.34). Among brain & CNS, bone, ovarian, and pancreatic cancer survivors, the increased risk ratio was not statistically significant between the lung cancer screening eligible and non‐eligible groups.

### 3.4 Risk of second primary lung cancer stratified by site of first primary cancer and lung cancer screening eligibility

For each cancer site, we stratified the risk of second primary lung cancer based on lung cancer screening eligibility status and compared it to the overall risk among all cancer survivors (i.e., all cancer sites combined), excluding lung cancer (Table 2).

**TABLE 2:** Risk of second primary lung cancer per cancer site compared to all cancer sites combined.

Group		2nd lung cancer	Total	Cum Inc.	Risk ratio	95% CI	Chi‐square	*p*‐value
All cancer sites, no lung		2144	185,934	0.0115	Reference	Reference		
*Cancer sites with high risk of 2nd primary lung cancer compared to the reference group*								
Head & Neck	All	305	9118	0.0335	2.90	2.58–3.26	336.831	<0.001
	Non‐eligible	134	6731	0.0199	1.73	1.45–2.05	39.014	<0.001
	Eligible	171	2387	0.0716	6.21	5.35–7.22	701.267	<0.001
Bladder	All	121	4552	0.0266	2.31	1.92–2.76	85.665	<0.001
	Non‐eligible	51	3352	0.0152	1.32	1.00–1.74	3.898	0.0483
	Eligible	70	1200	0.0583	5.06	4.01–6.37	223.392	<0.001
Lung	All	435	19,332	0.0225	1.95	1.76–2.16	169.877	<0.001
	Non‐eligible	159	10,338	0.0154	1.33	1.14–1.57	12.513	<0.001
	Eligible	276	8994	0.0307	2.66	2.35–3.01	256.766	<0.001
Cervical	All	53	2886	0.0184	1.59	1.22–2.09	11.540	<0.001
	Non‐eligible	33	2676	0.0123	1.07	0.76–1.51	0.148	0.7002
	Eligible	20	210	0.0952	8.26	5.43–12.56	127.916	<0.001
Breast	All	407	30,062	0.0135	1.17	1.06–1.30	8.938	0.0028
	Non‐eligible	249	28,035	0.0089	0.77	0.68–0.88	15.461	<0.001
	Eligible	158	2027	0.0779	6.76	5.79–7.90	731.163	<0.001
Prostate	All	283	21,614	0.0131	1.14	1.00–1.28	4.090	0.0431
	Non‐eligible	154	18,888	0.0082	0.71	0.60–0.83	17.633	<0.001
	Eligible	129	2726	0.0473	4.10	3.45–4.88	289.134	<0.001
*Cancer sites with similar risk of 2nd primary lung cancer compared to the reference group*								
Kidney & ureter	All	85	7877	0.0108	0.94	0.75–1.16	0.364	0.5463
	Non‐eligible	42	6640	0.0063	0.55	0.40–0.74	15.481	<0.001
	Eligible	43	1237	0.0348	3.01	2.24–4.06	57.425	<0.001
Colorectal	All	161	14,959	0.0108	0.93	0.80–1.09	0.720	0.3960
	Non‐eligible	92	13,227	0.0070	0.60	0.49–0.74	23.288	<0.001
	Eligible	69	1732	0.0398	3.45	2.73–4.37	117.998	<0.001
Lymphoma	All	117	10,960	0.0107	0.93	0.77–1.11	0.668	0.4138
	Non‐eligible	51	9944	0.0051	0.44	0.34–0.59	34.918	<0.001
	Eligible	66	1016	0.0650	5.63	4.44–7.14	246.939	<0.001
Melanoma	All	118	12,200	0.0097	0.84	0.70–1.01	3.505	0.0612
	Non‐eligible	79	11,203	0.0071	0.61	0.49–0.76	19.015	<0.001
	Eligible	39	997	0.0391	3.39	2.49–4.63	65.387	<0.001
Uterine	All	38	4242	0.0090	0.78	0.56–1.07	2.421	0.1197
	Non‐eligible	29	4009	0.0072	0.63	0.44–0.90	6.408	0.0114
	Eligible	9	233	0.0386	3.35	1.76–6.37	14.946	<0.001
Stomach	All	22	2638	0.0083	0.72	0.48–1.10	2.333	0.1266
	Non‐eligible	10	2309	0.0043	0.38	0.20–0.70	10.452	0.0012
	Eligible	12	329	0.0365	3.16	1.81–5.52	17.859	<0.001
Testicular	All	12	1517	0.0079	0.69	0.39–1.21	1.735	0.1878
	Non‐eligible	6	1468	0.0041	0.35	0.16–0.79	7.116	0.0076
	Eligible	6	49	0.1224	10.62	5.01–22.50	52.744	<0.001
Small intestine	All	12	1617	0.0074	0.64	0.37–1.13	2.383	0.1227
	Non‐eligible	8	1461	0.0055	0.47	0.24–0.95	4.682	0.0305
	Eligible	4	156	0.0256	2.22	0.84–5.86	2.720	0.0991
*Cancer sites with low risk of 2nd primary lung cancer compared to the reference group*								
Soft tissue	All	31	3861	0.0080	0.70	0.49–0.99	4.095	0.0430
	Non‐eligible	22	3609	0.0061	0.53	0.35–0.80	9.258	0.0023
	Eligible	9	252	0.0357	3.10	1.63–5.89	12.877	<0.001
Thyroid & parathyroid	All	42	5779	0.0073	0.63	0.46–0.85	9.037	0.0026
	Non‐eligible	34	5509	0.0062	0.54	0.38–0.75	13.663	<0.001
	Eligible	8	270	0.0296	2.57	1.30–5.09	7.731	0.0054
Leukemia	All	50	9302	0.0054	0.47	0.35–0.62	30.212	<0.001
	Non‐eligible	23	8447	0.0027	0.24	0.16–0.36	56.864	<0.001
	Eligible	27	855	0.0316	2.74	1.88–3.98	29.777	<0.001
Esophagus	All	25	3586	0.0070	0.60	0.41–0.90	6.464	0.0110
	Non‐eligible	9	2654	0.0034	0.29	0.15–0.57	15.362	<0.001
	Eligible	16	932	0.0172	1.49	0.91–2.42	2.579	0.1083
Liver & Biliary	All	14	3877	0.0036	0.31	0.19–0.53	21.194	<0.001
	Non‐eligible	7	3283	0.0021	0.18	0.09–0.39	25.357	<0.001
	Eligible	7	594	0.0118	1.02	0.49–2.14	0.003	0.9539
Pancreas	All	12	6101	0.0020	0.17	0.10–0.30	48.674	<0.001
	Non‐eligible	8	5123	0.0016	0.14	0.07–0.27	44.494	<0.001
	Eligible	4	978	0.0041	0.35	0.13–0.94	4.742	0.0294
Ovarian	All	22	7731	0.0028	0.25	0.16–0.38	50.628	<0.001
	Non‐eligible	18	7146	0.0025	0.22	0.14–0.35	50.479	<0.001
	Eligible	4	585	0.0068	0.59	0.22–1.58	1.128	0.2881
Bone	All	4	1603	0.0025	0.22	0.08–0.58	11.460	<0.001
	Non‐eligible	3	1504	0.0020	0.17	0.06–0.54	11.982	<0.001
	Eligible	1	99	0.0101	0.88	0.12–6.16	0.018	0.8940
Brain & CNS	All	3	6401	0.0005	0.04	0.01–0.13	68.603	<0.001
	Non‐eligible	2	5982	0.0003	0.03	0.01–0.12	65.710	<0.001
	Eligible	1	419	0.0024	0.21	0.03–1.47	3.072	0.0796

Abbreviations: Cum Inc.: Cumulative Incidence; 95% CI: 95% Confidence Interval.

#### 3.4.1 Cancer sites with high overall risk

**FIGURE 3:**
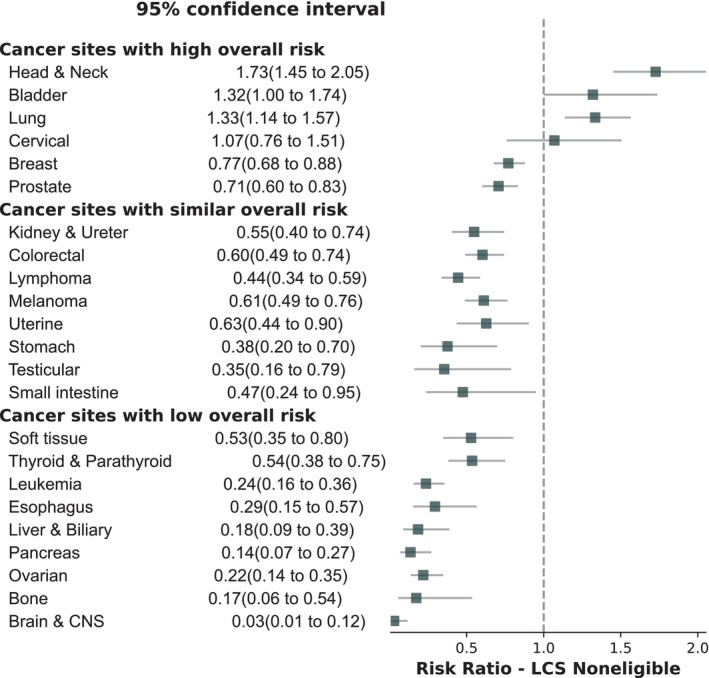
Risk of second lung cancer diagnosis in the lung cancer screening non‐eligible groups by First Primary Cancer Site relative to the overall risk (i.e., all cancers combined). LCS: Lung Cancer Screening.

**FIGURE 4:**
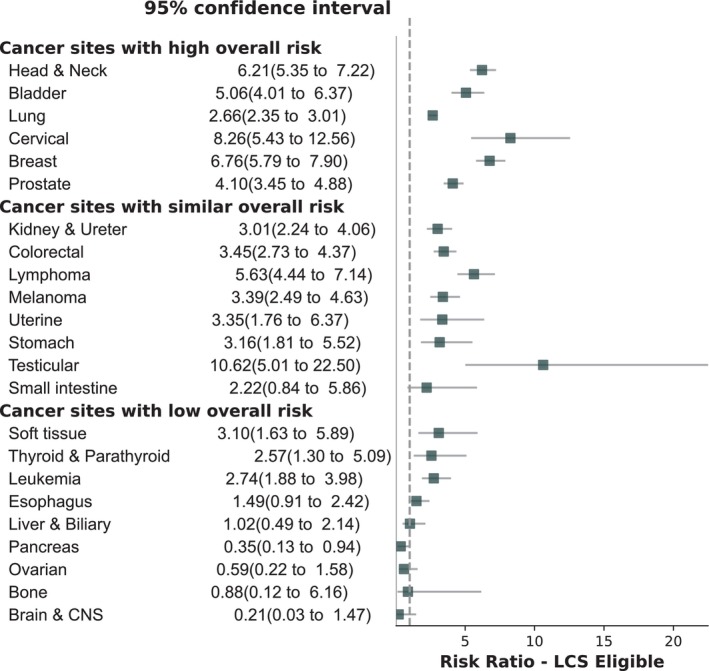
Risk of second lung cancer diagnosis in the lung cancer screening eligible groups by First Primary Cancer Site relative to the overall risk (i.e., all cancers combined). LCS: Lung Cancer Screening.

Among individuals with primary lung, H&N, bladder, or cervical cancer, the risks in the lung cancer screening non‐eligible groups remained higher with RRs ranging from 1.33 (95%CI:1.14–1.57, *p* < 0.001) in lung cancer to 1.73 (95%CI:1.45–2.05, *p* < 0.001) in H&N cancer (Figure 3). The increased risk among the lung cancer screening non‐eligible group in cervical cancer was not statistically significant. On the other hand, for breast and prostate cancer survivors, the risks among the lung cancer screening non‐eligible groups were significantly lower than the overall risk among all cancer survivors (Breast RR:0.77, 95%CI: 0.68–0.88; Prostate cancer RR:0.71, 95%CI:0.6–0.83). For lung, H&N, bladder, cervical, breast, and cervical cancer survivors, the risks in the lung cancer screening eligible groups were significantly higher than the overall risk among all cancer survivors. In the lung cancer screening eligible groups, RRs ranged between 2.66 (95%CI:2.35–3.01, *p* < 0.001) in lung cancer to 8.26 (95%CI:5.43–12.56, *p* < 0.001) in cervical cancer (Figure 4).

#### 3.4.2 Cancer sites with similar overall risk

For kidney & ureter, colorectal, lymphoma, melanoma, uterine, stomach, testicular, and small intestine cancer survivors, the risks were significantly lower among the lung cancer screening non‐eligible groups with RR ranging between 0.35 (95%CI:0.16–0.79, *p* = 0.0076) in testicular cancer and 0.63 (95%CI:0.44–0.9, *p* = 0.0114) in uterine cancer (Figure 3). Among lung cancer screening eligible groups, the risks were significantly higher with RR ranging between 10.62 (95%CI:5.01–22.5, *p* < 0.001) in testicular cancer and 3.01 (95%CI:2.24–4.06, *p* < 0.001) in kidney & ureter cancer, with the exception of small intestine cancer survivors where the increased risk was not statistically significant (Figure 4).

#### 3.4.3 Cancer sites with low overall risk

For soft tissue, thyroid & parathyroid, leukemia, esophagus, liver & biliary, pancreas, ovarian, bone, and brain & CNS cancer survivors, the risks of developing second primary lung cancer remained significantly lower in the lung cancer screening non‐eligible groups (Figure 3). Risk ratios ranged from 0.03 (95%CI: 0.01–0.12, *p* < 0.001) in brain & CNS cancer to 0.54 (95%CI:0.38–0.75, *p* < 0.001) in thyroid & parathyroid cancer. However, the risks among the lung cancer screening eligible groups significantly varied across those cancer sites as follows (Figure 4). For pancreas, ovarian, bone, and brain & CNS cancers, the risks remained lower than the overall risk for all cancer survivors but were not statistically significant except for pancreatic cancer survivors (RR:0.35, 95%CI:0.13–0.94, *p* = 0.0294). On the other hand, for soft tissue, thyroid & parathyroid, leukemia, esophagus, and liver & biliary cancers, the risks increased and were statistically significant except for esophagus and liver & biliary cancer survivors. The significant increased risk ratios ranged from 2.57 (95%CI: 1.3–5.09, *p* = 0.0054) in thyroid & parathyroid cancer to 3.1 (95%CI:1.63–5.89, *p* < 0.001) in soft tissue cancer.

### 3.5 Risk of second primary lung cancer stratified by its histology among cancer survivors non‐eligible for lung cancer screening

We found that among all cancer survivors non‐eligible for lung cancer screening, the proportion of adenosquamous carcinoma (ASC) was the highest (49%), followed by squamous cell carcinoma (SCC) 21%, adenocarcinoma (ADC) 14%, other non‐small cell lung cancer (NSCLC) 11%, and small cell lung cancer (SCLC) 5%. In addition, we compared the cumulative incidence of second primary lung cancer stratified by its histology among lung, H&N, and bladder cancer survivors non‐eligible for lung cancer screening. We found that among lung cancer survivors, the proportion of ASC was the highest (38%), followed by ADC 25%, SCC 19%, other NSCLC 12%, and SCLC 6%. Among H&N cancer survivors, the proportion of SCC was the highest (51%), followed by ASC 30%, other NSCLC 13%, SCLC 4%, and ADC 2%. Lastly, among bladder cancer survivors, the proportion of ASC was the highest (53%), followed by SCC 19%, ADC and other NSCLC 11%, and SCLC 6% (Table S2).

### 3.6 Risk of second primary lung cancer after the standard 5‐year surveillance period

We found that about half (49.6%) of cancer survivors diagnosed with second primary lung cancer were diagnosed after 5 years from their first primary cancer diagnosis. Most noteworthy, 54.3% of those individuals were not eligible for lung cancer screening. When looking at specific sites of first primary cancer, the proportions of lung, H&N, and bladder cancer survivors who developed second primary lung cancer after 5 years were 39.3%, 41%, and 42.2%, respectively. The proportions of those individuals who were not eligible for lung cancer screening were 42.9% in lung cancer, 48% in H&N cancer, and 39.2% in bladder cancer (Table S3).

## 4 DISCUSSION AND CONCLUSION

We evaluated the risk of second primary lung cancer among cancer survivors stratified by their lung cancer screening eligibility status using the 2021 USPSTF lung cancer screening recommendations. We found that cancer survivors with a personal history of lung, H&N, bladder, cervical, breast, and prostate cancers have significantly increased risk of second primary lung cancer compared to the overall risk of all cancer survivors (all cancer sites combined). When stratified by lung cancer screening eligibility status, lung, H&N, and bladder cancer survivors who are not eligible for lung cancer screening had statistically significantly higher risk of developing second primary lung cancer compared to all cancer survivors.

Our findings were consistent with prior research in which H&N,^13^ bladder,^19^, ^20^ cervical,^21^, ^22^ breast,^23^, ^24^ and prostate^25^, ^26^ cancer survivors had an increased risk of second lung, and ovarian^27^, ^28^ and soft tissue^29^ cancer survivors had a decreased risk, compared to the general population. In contrast, our results differed from previous findings for other primary cancer sites. While we found that melanoma, stomach, kidney, and uterine cancer survivors did not have a different risk of second lung cancer, prior research reported a decreased risk with melanoma^30^, ^31^ and stomach^32^ cancers and increased risk with kidney^33^ and uterine^34^, ^35^ cancers. Also, previous investigators identified esophageal cancer survivors to be at increased risk,^13^, ^36^, ^37^, ^38^ but our findings revealed a reduced risk of second lung cancer in this cohort. Prior literature reported conflicting results with the first primary testicular cancer, lymphoma, and leukemia. Testicular cancer survivors who received surgical treatment only had a decreased risk of second lung cancer, similar to our findings reported herein. However, the risk increased among those who received chemotherapy and/or radiotherapy.^39^ Also, the risk among lymphoma and leukemia cancer survivors significantly varied based on the treatment modality and the associated autoimmune conditions.^40^, ^41^, ^42^, ^43^ Of interest, while prior investigations compared the risk of lung cancer among cancer survivors with that of the general population, our methodology involved comparing the risk to that of all cancer survivors who already had an established increased risk of developing lung cancer. The different comparisons and the different nature of our study population, limited to those individuals 50 years or older, could explain the discrepancy in the results.

This study is the first to consider lung cancer screening eligibility status and stratify the risk of second lung cancer among cancer survivors based on both the site of the first primary cancer and the individual's lung cancer screening eligibility status. We found that across most cancer sites, the risk of second primary lung cancer among individuals who were eligible for lung cancer screening was significantly higher compared to individuals who were non‐eligible for lung cancer screening. This ascertains smoking history as an independent risk factor for developing second primary lung cancer. However, among ovarian, pancreas, bone, brain & CNS cancer survivors, the increased risk was not statistically significant, which might also suggest that the current eligibility criteria of the 2021 USPTSF screening guidelines might not be sufficient to explain the risk of second primary lung cancer in those cancer survivors.

Our findings suggest that other than lung cancer, personal history of H&N and bladder cancers, even those who are non‐eligible for lung cancer screening, are at increased risk of second primary lung cancer. This was consistent with our prior study among H&N cancer survivors.^13^ In addition, more than half of cancer survivors (51.4%) who developed second primary lung cancer were non‐eligible for lung cancer screening. Among those non‐eligible, NSCLC (ASC followed by SCC and ADC) was the most frequent histology of second primary lung cancer. This is important since studies have shown that lung cancer screening has higher sensitivity for NSCLC.^44^, ^45^ Furthermore, we found that significant numbers of all cancer survivors developed second primary lung cancer after the standard 5‐year surveillance period, and almost half of those individuals were not eligible for lung cancer screening. These findings highlight the importance of including personal history of cancer as an eligibility criterion for lung cancer screening since the benefits of screening extend beyond the surveillance period.

Our findings suggest that the current age and smoking history eligibility criteria of the 2021 USPTSF screening guidelines might not be sufficient to capture the risk of second lung cancer, especially among H&N and bladder cancer survivors. This is consistent with prior research that compared the performance of different eligibility criteria for lung cancer screening. More specifically, risk‐based eligibility criteria that select individuals for screening based on their personal risk calculated by a risk prediction model (e.g., the PLCOm2012 model) include, among other factors, personal history of cancer as an independent predictor and performed better than the current USPSTF criteria or the eligibility criteria used in the NELSON Trial.^46^, ^47^

This study has limitations. The self‐reported nature of smoking information can introduce the risk of recall bias. The diagnosis of second primary lung cancer was not pathologically confirmed; however, we defined an exclusion period of at least 6 months to distinguish between metastatic lung cancer and second primary lung cancer, an approach commonly used in similar studies. Our findings are based on data from one cancer institution, which can limit the generalizability of our conclusions due to differences in the patient characteristics receiving care at MDA as compared to the general population. Furthermore, the incidence of second primary lung cancer in MDA PHDB may be underestimated, as MDA patients are not followed for life, and other primary cancers would only be reported if patients returned to MDA for their second primary cancer. For these reasons, caution is warranted when generalizing the findings of this study to other populations.

Future research should incorporate factors such as sociodemographic data, treatment protocols, and follow‐up periods and investigate their potential impact on the relationship between personal history of cancer and the risk of second primary lung cancer based on screening eligibility. Also, future research is needed to optimize lung cancer screening at a more personalized level.^48^, ^49^, ^50^

In conclusion, we showed that personal history of H&N, bladder, cervical, breast, and prostate cancers is associated with increased risk of second primary lung cancer. H&N and bladder cancer survivors have a sufficiently high risk of developing second primary lung cancer, even in individuals who do not meet the 2021 USPSTF eligibility criteria. Personal history of H&N and bladder cancer warrants further consideration as an independent eligibility factor for lung cancer screening guidelines.

## AUTHOR CONTRIBUTIONS

**Sara Nofal:** Data curation; formal analysis; investigation; methodology; software; validation; visualization; writing – original draft; writing – review and editing. **Edwin J Ostrin:** Conceptualization; supervision; writing – review and editing. **Jianjun Zhang:** Conceptualization; funding acquisition; methodology; resources; supervision; writing – review and editing. **Jia Wu:** Conceptualization; supervision; writing – review and editing. **Paul Scheet:** Resources; supervision; writing – review and editing. **Mara B. Antonoff:** Conceptualization; supervision; writing – review and editing. **John V Heymach:** Conceptualization; funding acquisition; supervision; writing – review and editing. **Iakovos Toumazis:** Conceptualization; investigation; methodology; project administration; funding acquisition; resources; supervision; validation; writing – original draft; writing – review and editing.

## FUNDING INFORMATION

This study was supported by the National Cancer Institute grant R37CA271187 (PI: Toumazis) to Iakovos Toumazis; the generous philanthropic contributions to The University of Texas MD Anderson Cancer Center Lung Moon Shot to Jianjun Zhang. The funders did not play a role in the design of the study; the collection, analysis, and interpretation of the data; the writing of the manuscript; and the decision to submit the manuscript for publication.

## CONFLICT OF INTEREST STATEMENT

All authors have no conflicts of interest except the following: Sara Nofal reports support for the submitted work from the National Cancer Institute grant R37CA271187 (PI: Toumazis). Edwin Ostrin reports grant/contract by the Early Detection Research Network Clinical Validation Center (NCI) and payment/honoraria for Astra Zeneca (April 2021) outside the submitted work. He also reports having served on scientific advisory boards for Grail, Inc., and 20/20 GeneSystems, which have concluded as of December 2023. Jianjun Zhang reports grants from Merck, grants and personal fees from Johnson and Johnson and Novartis, personal fees from Bristol Myers Squibb, AstraZeneca, GenePlus, Innovent, and Hengrui outside the submitted work as well as support for the submitted work from the National Cancer Institute of the National Institute of Health Research Project Grant (R01CA234629), the AACR‐Johnson & Johnson Lung Cancer Innovation Science Grant (18‐90‐52‐ZHAN), and the MD Anderson Physician Scientist Program, MD Anderson Lung Cancer Moon Shot Program. Iakovos Toumazis reports support for the submitted work from the National Cancer Institute grant R37CA271187 (PI: Toumazis), and the generous philanthropic contributions to The University of Texas MD Anderson Cancer Center Lung Moon Shot (in part).

## ETHICS STATEMENT

This study was approved by the University of Texas M.D. Anderson Cancer Center International Review Board (IRB: 2021‐1158) and a waiver of consent was granted due to the retrospective nature of the study.

## Supporting information


**DATA S1.** Supporting Information.

## Data Availability

De‐identified data that support the findings of this study are available from the corresponding author upon reasonable request.
