# Supplementary material for: Risk of second primary lung cancer among cancer survivors stratified by the site of first primary cancer and the lung cancer screening eligibility status
Source: Int J Cancer. 2025 Apr 18;157(5):941–53. doi: 10.1002/ijc.35452 (PMC12232526; doi:10.1002/ijc.35452)

Title:

Risk of Second Primary Lung Cancer Among Cancer Survivors Stratified by The Site of First Primary Cancer and The Lung Cancer Screening Eligibility Status

Authors:

- Sara Nofal
- Edwin Ostrin
- Jianjun Zhang
- Jia Wu
- Paul Scheet
- Mara B. Antonoff
- John Heymach
- Iakovos Toumazis

Table of Contents

Supplementary Tables **2**

Supplementary Table 1. Risk of second primary lung cancer within each cancer site among lung cancer screening eligible compared to lung cancer screening non-eligible 2

Supplementary Table 2. Histology-specific cumulative incidence of second primary lung cancer among cancer survivors non-eligible for lung cancer screening in selected sites of first primary cancer 3

Supplementary Table 3. Cumulative incidence of second primary lung cancer based on the standard 5-year surveillance period stratified by screening eligibility status 4

Supplementary Figures **5**

Supplementary Figure 1. Frequency of First Primary Cancer by Site 5

Supplementary Table 1. Risk of second primary lung cancer within each cancer site among lung cancer screening eligible compared to lung cancer screening non-eligible

	**Screening Eligible**			**Screening Non-eligible**						
**1st Primary Cancer Site**	**2nd lung cancer**	**Total**	**Cum Inc**	**2nd lung cancer**	**Total**	**Cum Inc**	**Risk Ratio**	**95% CI**	**Chi-square**	**P-value**
**Lung**	276	8994	0.0307	159	10338	0.0154	2.00	1.64-2.42	51.235	<0.001
**Head & Neck**	171	2387	0.0716	134	6731	0.0199	3.60	2.88-4.49	145.846	<0.001
**Bladder**	70	1200	0.0583	51	3352	0.0152	3.83	2.69-5.47	63.493	<0.001
**Cervical**	20	210	0.0952	33	2676	0.0123	7.72	4.51-13.22	74.243	<0.001
**Breast**	158	2027	0.0779	249	28035	0.0089	8.78	7.23-10.66	675.162	<0.001
**Prostate**	129	2726	0.0473	154	18888	0.0082	5.80	4.61-7.31	282.832	<0.001
**Kidney & Ureter**	43	1237	0.0348	42	6640	0.0063	5.50	3.61-8.37	78.990	<0.001
**Colorectal**	69	1732	0.0398	92	13227	0.0070	5.73	4.21-7.79	155.533	<0.001
**Lymphoma**	66	1016	0.0650	51	9944	0.0051	12.67	8.84-18.15	312.461	<0.001
**Melanoma**	39	997	0.0391	79	11203	0.0071	5.55	3.80-8.10	98.276	<0.001
**Uterine**	9	233	0.0386	29	4009	0.0072	5.34	2.56-11.15	24.444	<0.001
**Stomach**	12	329	0.0365	10	2309	0.0043	8.42	3.67-19.34	35.976	<0.001
**Testicular**	6	49	0.1224	6	1468	0.0041	29.96	10.02-89.57	84.647	<0.001
**Small intestine**	4	156	0.0256	8	1461	0.0055	4.68	1.43-15.37	7.781	0.0053
**Soft tissue**	9	252	0.0357	22	3609	0.0061	5.86	2.73-12.59	25.945	<0.001
**Thyroid & Parathyroid**	8	270	0.0296	34	5509	0.0062	4.80	2.24-10.27	19.631	<0.001
**Leukemia**	27	855	0.0316	23	8447	0.0027	11.60	6.68-20.14	120.924	<0.001
**Esophagus**	16	932	0.0172	9	2654	0.0034	5.06	2.24-11.42	18.909	<0.001
**Liver & Biliary**	7	594	0.0118	7	3283	0.0021	5.53	1.95-15.70	13.025	<0.001
**Pancreas**	4	978	0.0041	8	5123	0.0016	2.62	0.79-8.68	2.674	0.1020
**Ovarian**	4	585	0.0068	18	7146	0.0025	2.71	0.92-7.99	3.554	0.0594
**Bone**	1	99	0.0101	3	1504	0.0020	5.06	0.53-48.24	2.452	0.1174
**Brain & CNS**	1	419	0.0024	2	5982	0.0003	7.14	0.65-78.56	3.521	0.0606

Cum Inc: Cumulative Incidence; 95% CI: 95% Confidence Interval

Supplementary Table 2. Histology-specific cumulative incidence of second primary lung cancer among cancer survivors non-eligible for lung cancer screening in selected sites of first primary cancer

**Histology**	**All cancer survivors**		**Lung cancer survivors**		**H&N cancer survivors**		**Bladder cancer survivors**	
	N	%	N	%	N	%	N	%
**NSCLC - ASC**	533	49.35%	55	37.67%	38	30.40%	25	53.19%
**NSCLC - ADC**	149	13.80%	36	24.66%	2	1.60%	5	10.64%
**NSCLC - SCC**	225	20.83%	28	19.18%	64	51.20%	9	19.15%
**NSCLC - other**	121	11.20%	18	12.33%	16	12.80%	5	10.64%
**SCLC**	52	4.81%	9	6.16%	5	4.00%	3	6.38%
**Total**	1080		146		125		47	

NSCLC: Non-small cell lung cancer; ASC: Adenosquamous carcinoma; ADC: Adenocarcinoma; SCC: Squamous cell carcinoma; and SCLC: small cell lung cancer

Supplementary Table 3. Cumulative incidence of second primary lung cancer based on the standard 5-year surveillance period stratified by screening eligibility status

	>=5-year surveillance			<5-year surveillance			Grand Total
	Screening Eligible n (%)[a]	Screening Non-eligible n (%)[a]	Total N (%)[b]	Screening Eligible n (%)[a]	Screening Non-eligible n (%)[a]	Total N (%)[b]	
**All cancer survivors**	542 (45.7)	644 (54.3)	1186 (49.6)	618 (51.2)	589 (48.8)	1207 (50.4)	2393
**Lung cancer survivors**	96 (57.1)	72 (42.9)	168 (39.3)	180 (69.2)	80 (30.8)	260 (60.8)	428
**H&N cancer survivors**	65 (52)	60 (48)	125 (41)	106 (58.9)	74 (41.1)	180 (59)	305
**Bladder cancer survivors**	31 (60.8)	20 (39.2)	51 (42.2)	39 (55.7)	31 (44.3)	70 (57.9)	121

[a] Row percentages are calculated based on the total for each surveillance period (>=5 years or < 5 years)

[b] Row percentages are calculated based on the grand total

Supplementary Figure 1. Frequency of First Primary Cancer by Site

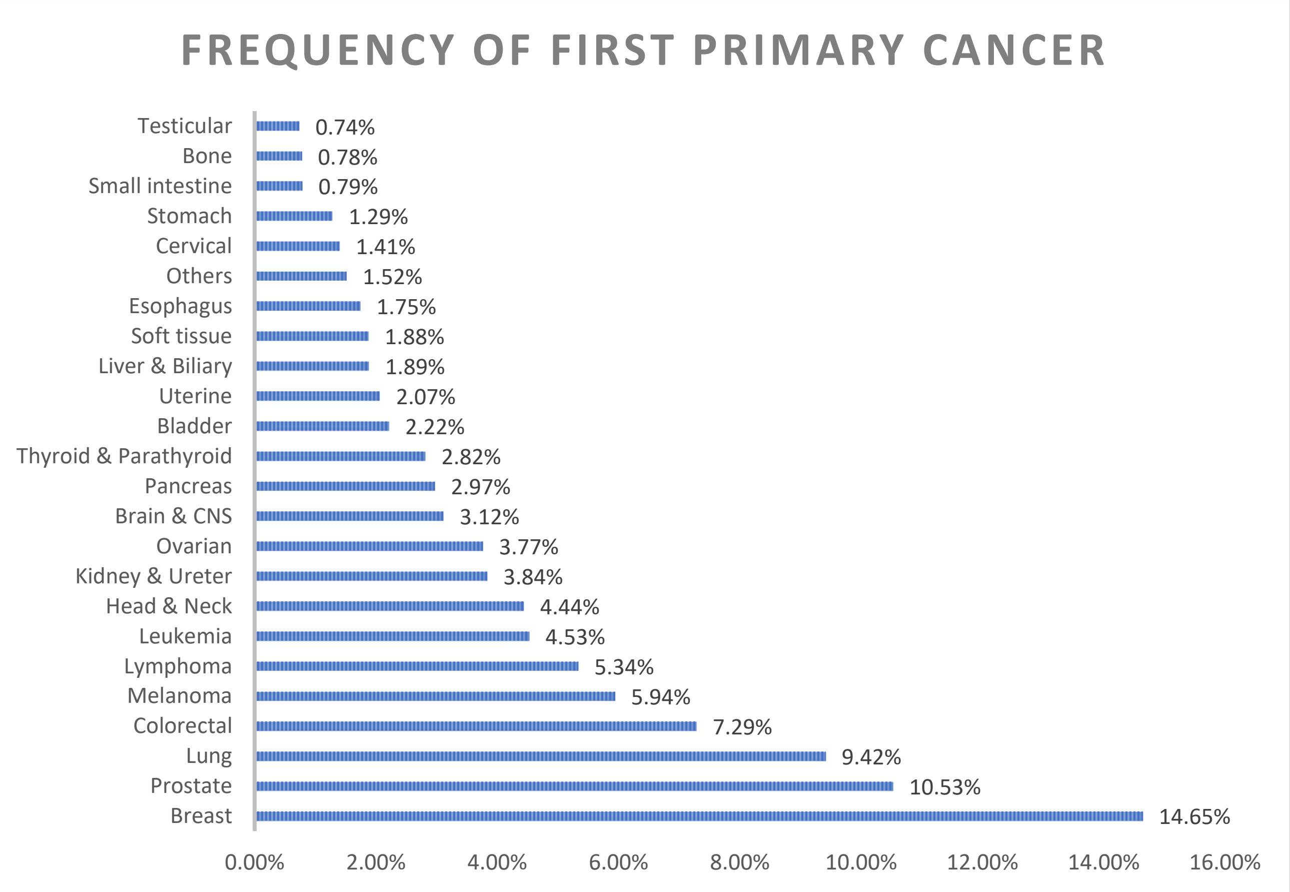

Supplement: Supplementary file 1 — DATA S1. Supporting Information. [file IJC-157-941-s001.pdf]
